# Anion and Cation Dynamics in Polyhydroborate Salts: NMR Studies

**DOI:** 10.3390/molecules25122940

**Published:** 2020-06-26

**Authors:** Alexander V. Skripov, Alexei V. Soloninin, Olga A. Babanova, Roman V. Skoryunov

**Affiliations:** Institute of Metal Physics, Ural Branch of the Russian Academy of Sciences, S. Kovalevskoi 18, 620108 Ekaterinburg, Russia; alex.soloninin@imp.uran.ru (A.V.S.); babanova@imp.uran.ru (O.A.B.); skoryunov@imp.uran.ru (R.V.S.)

**Keywords:** hydroborates, reorientations, rotational tunneling, diffusion, nuclear magnetic resonance

## Abstract

Polyhydroborate salts represent the important class of energy materials attracting significant recent attention. Some of these salts exhibit promising hydrogen storage properties and/or high ionic conductivities favorable for applications as solid electrolytes in batteries. Two basic types of thermally activated atomic jump motion are known to exist in these materials: the reorientational (rotational) motion of complex anions and the translational diffusion of cations or complex anions. The present paper reviews recent progress in nuclear magnetic resonance (NMR) studies of both reorientational and diffusive jump motion in polyhydroborate salts. The emphasis is put on sodium and lithium *closo*-borates exhibiting high ionic conductivity and on borohydride-based systems showing extremely fast reorientational motion down to low temperatures. For these systems, we discuss the effects of order–disorder phase transitions on the parameters of reorientations and diffusive jumps, as well as the mechanism of low-temperature rotational tunneling.

## 1. Introduction

Polyhydroborate salts are the ionic compounds described by a general formula *M_x_*[B*_m_*H*_n_*]*_y_*, where *M* is a metal cation and [B*_m_*H*_n_*] is a complex anion composed of boron and hydrogen atoms, such as [BH_4_]^−^, [B_10_H_10_]^2−^, or [B_12_H_12_]^2−^. These compounds represent the important class of energy materials attracting significant recent attention [1,2]. Light-metal tetrahydroborates based on the [BH_4_]^−^ anion are considered as promising materials for hydrogen storage due to their high volumetric and gravimetric hydrogen densities [3]; these compounds are often referred to as “borohydrides”. Another area of potential applications of hydroborate salts is related to their electrical conduction properties. It has been found recently that the disordered phases of some of *closo*-hydroborate salts, such as *M*_2_B_12_H_12_ and *M*_2_B_10_H_10_ (*M* = Na, Li), exhibit extremely high ionic conductivities [4,5,6,7]. Furthermore, these materials appear to be very stable; therefore, they are considered as prospective solid electrolytes for Li- and Na-ion batteries [8,9,10,11].

The important dynamical feature of hydroborate salts is that complex anions can participate in the fast reorientational (rotational) motion [12,13]. This motion strongly contributes to the balance of energies determining thermodynamic stability; therefore, information on the anion reorientational dynamics is crucial for understanding the fundamental properties of hydroborate salts. Apart from the localized reorientational motion of the anions, in compounds showing high ionic conductivities, the long-range diffusive motion of the cations should also be prominent. Moreover, these two types of atomic motion may be related. Indeed, a number of experimental studies have revealed that the fast diffusive motion of the cations in hydroborate salts is accompanied by the fast reorientational motion of the anions [5,7,14,15,16,17]. Recent ab initio molecular dynamics calculations strongly suggest that reorientations of large *closo*-hydroborate anions can facilitate cation mobility [18,19,20,21]. Thus, understanding the anion and cation dynamics in hydroborate salts may give a key to improving their application-related properties. Nuclear magnetic resonance (NMR) has proved to be an efficient technique for studies of atomic motion in solids at the microscopic level. This technique was widely used to investigate the atomic jump motion in alkali–metal borohydrides [12,13,14,15,22,23,24,25,26,27] and alkaline–earth borohydrides [28,29,30,31]. However, systematic studies of the anion and cation dynamics in more complex systems, such as mixed BH_4_-based compounds and *closo*-hydroborates and the related compounds, have just begun. It should be noted that the search for ionic conductivity in *closo*-hydroborates has been initiated by the NMR results [17] revealing that the transition from the ordered low-temperature phase of Na_2_B_12_H_12_ to the disordered high-temperature phase is accompanied by the abrupt (nearly two orders of magnitude) acceleration of both anion reorientations and cation diffusion, and that the Na^+^ jump rate just above the transition temperature exceeds 10^8^ s^−1^. This work presents a brief review of recent progress in NMR investigations of anion and cation dynamics in hydroborate salts. The emphasis is put on sodium and lithium *closo*-hydroborates and the related systems exhibiting high ionic conductivity and on borohydride-based systems showing extremely fast reorientational motion down to low temperatures. For these systems, we discuss the relation between the anion reorientations and the cation diffusion, the effects of the order–disorder phase transitions on the motional parameters, as well as the mechanism of low-temperature rotational tunneling.

## 2. NMR Approach to Dynamical Studies

The applications of nuclear magnetic resonance to studies of atomic motion are based on the sensitivity of NMR parameters to fluctuations of local magnetic and electric fields arising due to atomic jumps. The range of atomic jump rates that can be probed by the standard NMR techniques is of the order of 10^4^–10^11^ s^−1^. In some cases, the use of such special NMR techniques as measurements of the decay of dipolar spin order [32] and spin-alignment echo spectroscopy [33] may allow one to probe ultraslow atomic motion with characteristic jump rates in the range of 10^−1^–10^3^ s^−1^.

The most easily measured parameter is the width of the NMR spectrum. For proton (^1^H) NMR, the low-temperature value of the line width, Δ_HR_, is usually determined by static dipole–dipole interactions between nuclear spins. For hydroborates, typical values of such a “rigid lattice” line width are of the order of 10^4^–10^5^ s^−1^. If H atoms are involved in thermally activated jump motion, the line width starts to decrease with increasing temperature due to the averaging of dipole–dipole interactions between nuclear spins. The onset of this motional line narrowing occurs when the H jump rate *τ*^−1^ becomes nearly equal to Δ_HR_ [34]. In the case of long-range diffusion of H atoms (or H-containing species), the dipole–dipole interactions are completely averaged out when *τ*^−1^ » Δ_HR_, and at high temperatures, the line width drops to very small values determined mostly by the magnetic field inhomogeneity. However, in the case of localized motion (such as anion reorientations), the dipole–dipole interactions are not fully averaged out even in the limit of *τ*^−1^ » Δ_HR_: “intramolecular” interactions (between nuclear spins within the same reorienting anion) are usually averaged out, while “intermolecular” interactions (between nuclear spins on different reorienting anions) are averaged only partially. In this case, at high temperatures, the line width exhibits a plateau with the level corresponding to a substantial fraction of Δ_HR_. Thus, the behavior of the ^1^H NMR line width at high temperatures allows us to distinguish the cases of localized motion and long-range diffusion of H atoms. Similar qualitative features of NMR line narrowing are also expected for other nuclei participating in the jump motion. However, for nuclei with nonzero electric quadrupole moment (those with the spin *I* > ½, such as ^7^Li and ^23^Na), we should take into account the combined effect of dipole–dipole interactions between nuclear spins and interactions between the nuclear electric quadrupole moments and local electric field gradients. It should be noted that measurements of the NMR line width can trace the changes in the atomic jump rate over a rather limited dynamic jump range (typically, not exceeding two orders of magnitude). In order to probe a much broader dynamic range, it is necessary to measure the nuclear spin–lattice relaxation rate *R*_1_ characterizing the recovery of nuclear spin magnetization after deviations of a nuclear spin system from the equilibrium state.

The measured nuclear spin–lattice relaxation rate *R*_1_ in solids may contain a number of additive contributions of different physical nature. However, for most of the studied hydroborate-based systems, the dominant relaxation mechanism of ^1^H nuclei is due to dipole–dipole interactions modulated by atomic jump motion. For nuclei with *I* > ½, the electric quadrupole interaction modulated by atomic motion may also be important. The characteristic feature of the motional contribution to *R*_1_(*T*) is a maximum at the temperature at which the atomic jump rate *τ*^−1^ becomes nearly equal to the (circular) nuclear magnetic resonance frequency *ω*, i.e., when *ωτ* ≈ 1 [34]. Since typical values of *ω* are of the order of 10^8^–10^9^ s^−1^, the *R*_1_(*T*) peak corresponds to much higher atomic jump rates than the onset of NMR line narrowing. The amplitude of the *R*_1_(*T*) peak is determined by the strength of dipole–dipole and/or quadrupole interactions that are caused to fluctuate due to atomic motion; in addition, this amplitude is inversely proportional to *ω*. Therefore, it is often advantageous to use spin–lattice relaxation measurements at low resonance frequencies (i.e., at low magnetic fields).

In the limit of slow atomic motion (*ωτ* » 1), the motional contribution to *R*_1_ is proportional to *ω*^−2^*τ*^−1^, and in the limit of fast motion (*ωτ* « 1), the motional contribution to *R*_1_ is proportional to *τ* being frequency-independent. If the temperature dependence of *τ* follows the Arrhenius law, *τ* = *τ*_0_exp(*E*_a_/*k*_B_*T*), where *E*_a_ is the activation energy, a plot of ln*R*_1_ vs. *T*^−1^ is expected to be linear in the limits of both slow and fast motion with the slopes of −*E*_a_/*k*_B_ and *E*_a_/*k*_B_, respectively. Thus, the activation energy for the atomic motion can be obtained directly from these slopes. Apart from these limiting cases, the relation between the motional contribution to *R*_1_ and the atomic jump rate is usually based on certain models. For long-range atomic diffusion, the most widely used model is that introduced by Bloembergen, Purcell, and Pound (BPP) [35]. The BPP model correctly describes the main features of the motional contribution to *R*_1_ (including the asymptotic behavior in the limits of slow and fast motion) and provides relatively simple expressions relating *R*_1_ and *τ*. For some simple types of reorientational motion, the relation between *R*_1_ and *τ* can be calculated analytically [34,36], and in many cases, the corresponding expressions are analogous to those given by the BPP model. However, the amplitudes of the *R*_1_(*T*) peaks resulting from reorientations are usually lower than in the case of long-range diffusion, since localized motion only partially modulates the “rigid lattice” dipole–dipole (or quadrupole) interactions. It should be emphasized that the motional contribution to *R*_1_ depends on *τ nonmonotonically*. This feature allows one to probe the atomic jump rates over much broader dynamic ranges than the range of the measured *R*_1_ values themselves. In favorable cases, it is possible to trace the changes in *τ*^−1^ over the dynamic range of eight orders of magnitude (10^4^–10^12^ s^−1^), as it was demonstrated for BH_4_ reorientations in NaBH_4_ and KBH_4_ [23]. If considerable distributions of *τ*^−1^ values are present (as typical, for example, of disordered systems), the observed low-*T* slope of the *R*_1_(*T*) peak becomes less steep than the high-*T* one, and the frequency dependence of *R*_1_ at the low-*T* slope becomes weaker than *ω*^−2^ [37]. Therefore, spin–lattice relaxation measurements over wide ranges of temperature and the resonance frequency give an opportunity to reveal the presence of jump rate distributions.

Besides NMR techniques giving information on the atomic jump rates, there is also the unique NMR method–spin echo with pulsed-field gradient (PFG) [38,39,40]–that allows one to measure the tracer diffusion coefficients directly. This method provides a bridge between microscopic and macroscopic measurements of atomic mobility. It is based on observation of an additional attenuation of the spin echo signal due to the displacement of nuclear spins in an external magnetic field gradient. In most PFG-NMR experiments, the field gradient is applied in the form of two rectangular pulses; the time interval between the two gradient pulses is usually fixed, and the echo intensity is studied as a function of the gradient pulse amplitude *g*. The application of the field gradient serves to set a spatial scale in a certain direction. The echo attenuation is simply related to the displacement of nuclear spins along the direction of the gradient during the time interval between the gradient pulses. Therefore, the PFG-NMR technique represents a direct way of measuring the tracer diffusion coefficient *D*. The range of atomic or molecular diffusion coefficients that can be measured by the PFG-NMR in solids is typically 10^−8^–10^−4^ cm^2^/s. The lower limit of the measurable *D* range is determined mainly by the maximum *g* values available from the pulse circuitry and by the spin–spin relaxation rates (or the spin–lattice relaxation rates in case of using the stimulated echo sequence [41]) responsible for intrinsic attenuation of the spin echo signal. Modern PFG-NMR spectrometers can generate pulsed gradients with the amplitudes up to ~3 kG/cm. Typical intervals between the gradient pulses (diffusion times) are of the order of 10 ms. If the tracer diffusion coefficient is 10^−6^ cm^2^/s, the mean displacement of an atom for this time is of the order of 2 × 10^−4^ cm. Thus, the PFG-NMR technique can probe the diffusivity over the spatial scale of a few microns. Comparison of the experimental results for the diffusivity with those for the atomic jump rates *τ*^−1^ can give useful information on the diffusion path. Indeed, neglecting any correlations for diffusive jumps, the elementary jump length *L* can be estimated from the expression *D* = *L*^2^ *τ*^−1^/6 (in the case of three-dimensional diffusion).

It is often advantageous to combine NMR experiments with incoherent quasielastic neutron scattering (QENS) measurements [42,43], since these two methods are known to give complementary microscopic information on atomic jump motion in materials. It is worth noting that nuclear spins play a crucial role in both methods. While for NMR this is quite evident, one should also keep in mind that the incoherent part of neutron scattering by nuclei of the same isotope is determined by the difference between the scattering lengths for different mutual spin orientations of a neutron and a nucleus. For isotopes with a zero nuclear spin (such as ^12^C and ^16^O), the incoherent neutron scattering cross-section is also zero. The incoherent neutron scattering cross-section for protons (^1^H) is more than an order of magnitude higher than for all other isotopes; therefore, the scattering from H-containing materials is usually dominated by the contribution due to protons. Because of this feature, QENS is quite effective for studies of the jump motion of H atoms and H-containing groups; however, its application to the motion of other atoms in complex hydrides (for example, metal cations in hydroborates) is problematic. Another problem typical of hydroborates is related to the very large neutron absorption cross-section of ^10^B isotope with a natural abundance of 19.6%. This problem can be lifted by using ^11^B-enriched starting materials for the synthesis of hydroborates. Incoherent QENS measurements can probe the atomic jump rates over the dynamic range of ~10^8^–10^12^ s^−1^. The lower limit of this range is determined by the energy resolution of the available neutron spectrometers. Thus, the ranges of the atomic jump rates probed by NMR and QENS are partially overlapping, and NMR has evident advantages in terms of the width of the dynamic range, being sensitive to much slower motion than QENS. However, standard NMR measurements usually cannot give information on the spatial aspects of atomic motion. Indeed, because of their local nature, the measured NMR parameters are represented as integrals over the entire *Q*-space. In contrast, incoherent QENS measurements can probe the *Q* dependences of the autocorrelation functions [42,43], giving information on the geometry of atomic jumps. The usual approach is based on a comparison of the experimental *Q* dependence of QENS spectra with that calculated for various geometrical models of atomic jump motion [42,43]. Such an approach appears to be effective, if QENS measurements are performed over a wide range of the neutron momentum transfer *ħQ* (at least, up to *Q* ≈ 3 Å^−1^).

## 3. Ultrafast Low-Temperature Reorientational Motion of BH_4_ Anions

In a number of BH_4_-based compounds, the anions were found to retain very high reorientational mobility down to low temperatures. For these systems, the proton spin–lattice relaxation rate maximum is observed below 80 K; therefore, the reorientational jump rate *τ*^−1^ reaches the values of about 10^8^ s^−1^ at the temperature of the maximum. This implies that the energy barriers for the reorientational motion should be very low. The archetypical example of such systems is represented by LiBH_4_-LiI solid solutions. A partial halide ion substitution of BH_4_^−^ anions in lithium borohydride is found [44] to stabilize the structure of the high-*T* (hexagonal) phase of LiBH_4_ down to low temperatures. The temperature dependence of the ^1^H spin–lattice relaxation rate $R_{1}^{H}$ in the solid solutions Li(BH_4_)_1-*y*_I*_y_* (*y* = 0.33, 0.5, 0.67) exhibits the frequency-dependent low-temperature maximum [45]. At the frequency of 14 MHz, this maximum is observed near 55 K for *y* = 0.33, 40 K for *y* = 0.5, and 33 K for *y* = 0.67 [45]. Thus, the reorientational motion of BH_4_^−^ anions becomes faster with increasing iodine content. This trend is consistent with the results of QENS experiments for Li(BH_4_)_1-*y*_I*_y_* (*y* = 0.2, 0.33, 0.5) at *T* ≥ 200 K [46]. Since the ionic radius of I^−^ is larger than that of BH_4_^−^, and the increase in I^−^ content leads to the increase in the lattice parameters of the solid solution, it is natural to attribute the acceleration of reorientational motion at higher I^−^ concentrations to lower energy barriers for BH_4_^−^ reorientations due to weaker H–Li interactions. It should be noted that the shape of the low-temperature $R_{1}^{H}(T)$ peak in Li(BH_4_)_1-*y*_I*_y_* is quite complex; it can be satisfactorily described as a superposition of two partially overlapping peaks [45]. This suggests the coexistence of two reorientational jump processes with different motional parameters. The nature of the two reorientational processes has been clarified by QENS measurements for Li(BH_4_)_0.5_I_0.5_ [47]. These experiments performed over a wide range of the neutron momentum transfer *ħQ* have revealed that the faster jump process corresponds to reorientations around the three-fold symmetry axis (directed along the crystallographic *c* axis) of the BH_4_ tetrahedron. Such a process leaves one of the four H atoms immobile (the one located at the three-fold reorientational axis of the BH_4_ group). The slower jump process has been attributed to the jump exchange between one of the three rotating H atoms and the static axial H atom [47].

**Figure 1:**
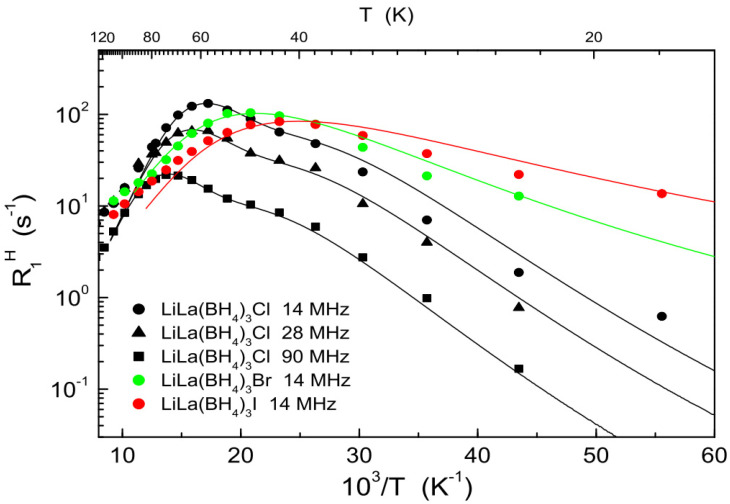
The behavior of the ^1^H spin–lattice relaxation rates in LiLa(BH_4_)_3_Cl [52], LiLa(BH_4_)_3_Br, and LiLa(BH_4_)_3_I [53] at low temperatures. The relaxation rates measured at different resonance frequencies are shown as functions of the inverse temperature. For LiLa(BH_4_)_3_Br and LiLa(BH_4_)_3_I, only the results at 14 MHz are included in the figure. The curves show the simultaneous fits of the two-peak model to the data for LiLa(BH_4_)_3_Cl (black lines) and the model with a Gaussian distribution of activation energies to the data for LiLa(BH_4_)_3_Br and LiLa(BH_4_)_3_I (green and red lines, respectively).

Another interesting family of BH_4_-based compounds showing fast low-temperature reorientational motion is represented by bimetallic borohydrides-halides Li*R*(BH_4_)_3_*X*, where *R* = La, Ce, Gd, and *X* = Cl, Br, I [48,49,50,51]. The unusual cubic structure of these isomorphous compounds (space group *I*4−3*m*) consists of isolated anionic clusters [*R*_4_*X*_4_(BH_4_)_12_]^4−^ with a distorted cubane *R*_4_*X*_4_ core, charge-balanced by Li^+^ cations. Each *R* atom is coordinated by three *X* atoms and three BH_4_ groups via the H_3_ face, and Li^+^ ions randomly occupy 2/3 of the available 12*d* sites. Since localized magnetic moments on Ce and Gd strongly affect the measured ^1^H spin–lattice relaxation rates, NMR studies of BH_4_ reorientations have proved to be possible only in compounds with *R* = La [52]. The low-temperature reorientational motion of BH_4_ groups in all LiLa(BH_4_)_3_*X* compounds is found to be very fast. Figure 1 shows the low-temperature behavior of the ^1^H spin–lattice relaxation rates for LiLa(BH_4_)_3_*X* (*X* = Cl, Br, I) [52,53]. At the resonance frequency of 14 MHz, the relaxation rate maximum is observed near 58 K for LiLa(BH_4_)_3_Cl, 48 K for LiLa(BH_4_)_3_Br, and 43 K for LiLa(BH_4_)_3_I. These results indicate that the low-temperature BH_4_ reorientations become faster with the increase in the halide ionic radius (and the related increase in the lattice parameter [51]). The local environment of BH_4_ groups suggests that the fast motional process corresponds to reorientations around the three-fold symmetry axis directed along the La–B line [52]. Both the temperature and frequency dependences of $R_{1}^{H}$ for LiLa(BH_4_)_3_*X* in the low-*T* region are well described in terms of the models with Gaussian distributions of the activation energies (two-peak distribution for *X* = Cl, and one-peak distributions for *X* =Br and I). The presence of distributions of the motional parameters in these systems appears to be quite natural. Indeed, because of the random occupancy of two-thirds of the available 12*d* sites by Li^+^ ions, the local environment of BH_4_ groups changes from one group to another.

The fast low-temperature reorientational motion of BH_4_^−^ has also been revealed in the mixed borohydride-amide Na_2_(BH_4_) (NH_2_) [54] having the cubic antiperovskite-type structure (space group *Pm*-3*m*) [55,56]. For this compound, the proton spin–lattice relaxation rate maximum at the frequency of 14 MHz is observed near 70 K, and in the temperature range 13–209 K the experimental $R_{1}^{H}(T)$ data at three resonance frequencies are satisfactorily described by the model with a Gaussian distribution of the activation energies [54]. As in the case of LiLa(BH_4_)_3_*X* compounds, the presence of the activation energy distribution in Na_2_(BH_4_)(NH_2_) can be related to the spread in local environments of BH_4_ groups due to the random occupancy of two-thirds of the available 3*d* sites by Na^+^ ions.

**Figure 2:**
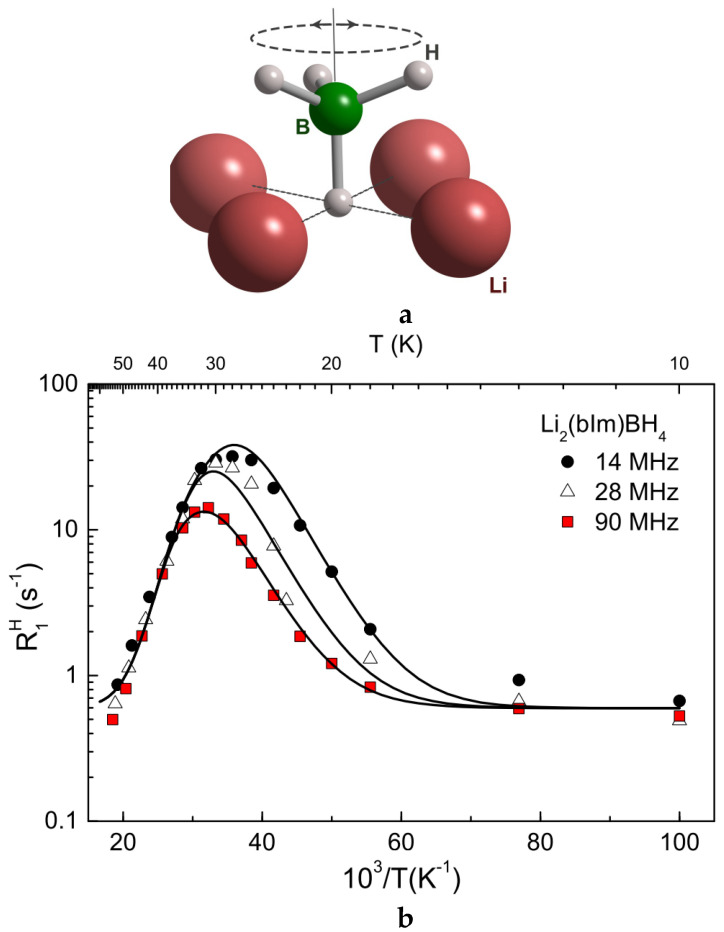
(**a**) Schematic view of the local coordination of each BH_4_^−^ anion in Li_2_(bIm)BH_4_. This coordination allows facile reorientations of three H atoms (as depicted by the dashed ellipse) around the single B-H bond axis [60]. (**b**) Low-temperature proton spin–lattice relaxation rates measured at 14, 28, and 90 MHz as functions of the inverse temperature. Solid lines show the simultaneous fit of the model with rotational tunneling to the data. Reprinted with permission from [60]. Copyright 2019 American Chemical Society.

As noted above, the occurrence of fast low-temperature BH_4_^−^ reorientations in some borohydrides suggests low energy barriers for the reorientational motion (of the order of 50 meV or less [16]), which are somewhat unusual for ionic compounds. Low energy barriers for reorientations may lead to the low-temperature rotational tunneling that has been extensively studied for CH_3_ and NH_4_ groups [57,58]. However, until recently, it was not clear, whether the mechanism of rotational tunneling can be observed for BH_4_^−^ anions in borohydrides. This possibility has been considered for the ammine-borohydride Sr(BH_4_)_2_(NH_3_)_2_, where the small-amplitude $R_{1}^{H}(T)$ peak is found near 15 K [59]. However, a comparison of the measured ^1^H and ^11^B spin–lattice relaxation rates suggests that this peak originates from NH_3_ reorientations [59]. The first direct evidence of the rotational tunneling of BH_4_^−^ anions was obtained in the recent study [60] combining NMR and QENS measurements for lithium benzimidazolate-borohydride Li_2_(bIm)BH_4_ (where the benzimidazolate anion, C_7_N_2_H_5_^−^, is denoted by bIm^−^). The local environment of BH_4_^−^ anions in the low-temperature monoclinic structure of Li_2_(bIm)BH_4_ (space group *C*2/*m* [60]) appears to be quite unusual: one H atom of the BH_4_ group is anchored within a nearly square hollow formed by four coplanar Li^+^ cations, while the remaining −BH_3_ fragment extends into a relatively open space, being only loosely coordinated to other atoms (see Figure 2a). Such open BH_4_ coordination suggests the possibility of facile rotations around the anchored B–H bond (three-fold symmetry axis). 

Figure 2b shows the low-temperature behavior of the ^1^H spin–lattice relaxation rates measured at three resonance frequencies for Li_2_(bIm)BH_4_. At the frequency of 14 MHz, the relaxation rate maximum is observed near 28 K. The $R_{1}^{H}$ leveling-off toward a constant plateau below 16 K can be attributed to an additional “background” contribution due to spin diffusion to paramagnetic impurities [61]. It should be noted that the behavior of $R_{1}^{H}$ in the region of the peak significantly deviates from that predicted by the standard theory for thermally activated atomic motion. In particular, the frequency dependence of the relaxation rate near the peak appears to be considerably weaker than that predicted by the standard theory. Furthermore, the measured ^1^H NMR line width at 6 K is much smaller than that estimated on the basis of “rigid lattice” calculations for Li_2_(bIm)BH_4_, and it does not show any significant changes over the wide temperature range (6–298 K) [60]. These features are typical of the case of rotational tunneling [62]. Comparison of the observed frequency dependence of $R_{1}^{H}$ near the peak with the corresponding results for methyl group rotations in various compounds [63] suggests that for Li_2_(bIm)BH_4_, the tunneling frequency *ω_t_* (that determines the librational ground-state splitting *ħω_t_*) should be of the order of the resonance frequency *ω*. The solid lines in Figure 2 show the simultaneous fit of the $R_{1}^{H}(T)$ data at three resonance frequencies to the model [63] describing a smooth transition from quantum dynamics at low temperatures to classical jump motion at higher temperatures. In terms of this model, the low-*T* slope of the $R_{1}^{H}(T)$ peak is determined by the energy difference between the librational ground-state and the first excited state, *E*_01_, while the high-*T* slope is determined by the classical activation energy *E*_a_ related to the potential barrier height. The most important energy parameters resulting from the fit are *ω_t_* = 7.5(3) × 10^8^ s^−1^ (corresponding to the tunnel splitting *ħω_t_* of 0.49 μeV), *E*_01_ = 17.7(8) meV, and *E*_a_ = 44.1(2) meV.

Thus, NMR results for Li_2_(bIm)BH_4_ are consistent with the low-temperature rotational tunneling of BH_4_^−^ anions; however, unambiguous evidence of the tunnel splitting can be obtained only from inelastic neutron scattering experiments with high energy resolution: in addition to the quasielastic peak centered at zero neutron energy transfer, the neutron spectrum is expected to contain some extra peaks at finite energy transfer values corresponding to the energy differences between the tunnel-split librational levels [57,58].

The most effective approach to measuring the tunnel splitting of less than 1 μeV is based on the neutron spin echo (NSE) technique [64] that directly probes the neutron energy changes in the scattering process using the neutron spin precession period in a magnetic field as the internal clock. This technique provides the best energy resolution among the available neutron spectroscopic methods. The quantity measured by NSE is proportional to the intermediate scattering function *I*(*Q*,*t*) [42,64] that represents the time Fourier transform of the spectral neutron scattering function. If extra peaks at finite energy transfers are present in the neutron spectrum, *I*(*Q*,*t*) is expected to show oscillatory behavior as a function of time. For Li_2_(bIm)BH_4_, such an oscillatory behavior of *I*(*Q*,*t*) has been found at low temperatures (3.6 K and 20 K) [60], yielding the tunnel splitting *ħω_t_* of 0.43(2) μeV at 3.6 K. However, at 30 K, the oscillatory behavior is no longer observable, so that *I*(*Q*,*t*) becomes a monotonically decreasing function of time. This indicates a gradual transition to the regime of classical jump reorientations at higher temperatures. The *Q* dependence of QENS spectra in the classical jump regime (80 K) is consistent with the model of uniaxial BH_4_ reorientations around the three-fold symmetry axis [60], as suggested by the geometry of local environment of the BH_4_ groups in Li_2_(bIm)BH_4_. Summarizing the results of combined NMR and neutron scattering studies of the dynamical properties of Li_2_(bIm)BH_4_ [60], we can conclude that this compound exhibits the first well-documented case of the low-temperature rotational tunneling of BH_4_^−^ anions. It seems probable that the rotational tunneling may also be found in other complex borohydrides, where BH_4_ groups are somewhat isolated from other structural units.

## 4. Anion Reorientations and Cation Diffusion in B_12_H_12_- and B_10_H_10_-Based *closo*-Borates and Related Compounds 

The first studies of reorientational anion motion in *closo*-dodecaborates were reported for the isomorphous cubic compounds *A*_2_B_12_H_12_ (*A* = K^+^, Rb^+^, NH_4_^+^, Cs^+^) [65]. On the basis of the observed narrowing of the ^11^B NMR spectra, it has been found that the [B_12_H_12_]^2−^ anions in these compounds participate in the reorientational motion, and the jump rate of the reorientations increases with increasing cation radius. For the cubic *closo*-dodecaborate with the fastest reorientations, Cs_2_B_12_H_12_, the reorientational motion has also been investigated by QENS [66]. The *Q* dependence of QENS spectra for Cs_2_B_12_H_12_ is consistent with the model of jump reorientations of [B_12_H_12_]^2−^ around a single symmetry axis (either five-fold or three-fold) at 430 K, while at temperatures of 480 K and higher, the model of reorientations around two symmetry axes appears to be preferable. The systematic measurements of the temperature and frequency dependences of the ^1^H spin–lattice relaxation rates for K_2_B_12_H_12_, Rb_2_B_12_H_12_, Cs_2_B_12_H_12_ [17], and for (NH_4_)_2_B_12_H_12_ [67] performed over wide dynamic ranges of jump rates have yielded accurate values of the activation energies for [B_12_H_12_]^2−^ reorientations. These values are included in Table 1 which summarizes the activation energies for anion reorientations in *closo*-borates and related compounds. While the $R_{1}^{H}(T)$ results [17,67] for the series *A*_2_B_12_H_12_ (*A* = K^+^, Rb^+^, NH_4_^+^, Cs^+^) support the conclusion that the reorientations become faster with increasing cation radius, they also indicate that the activation energies derived from the ^11^B NMR line width [65] are considerably overestimated. Indeed, as discussed above in Section 2, NMR line width measurements can only trace the changes in jump rates over a narrow dynamic range. An additional indication of the overestimated *E*_a_ values in [65] are the unreasonably small pre-exponential factors *τ*_0_ (< 10^-21^ s) obtained in this work. 

**Figure 3:**
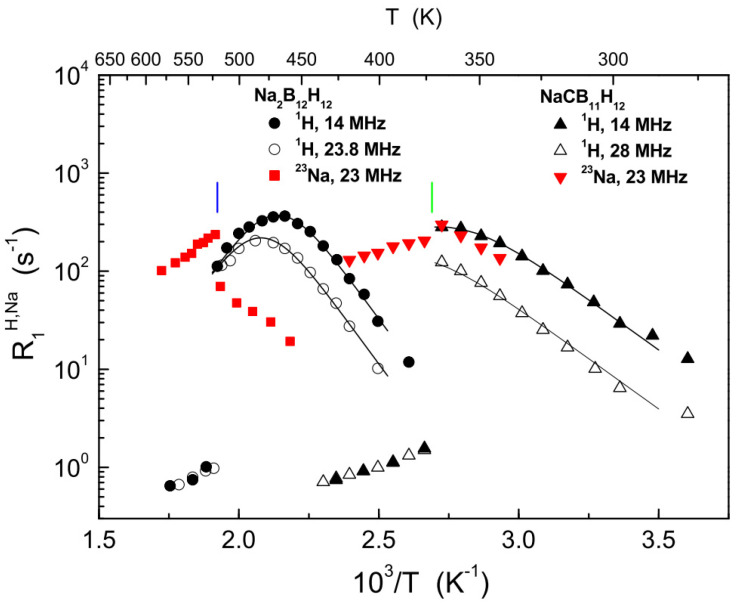
Effects of the order–disorder phase transitions in Na_2_B_12_H_12_ [17] and NaCB_11_H_12_ [71] on the ^1^H and ^23^Na spin–lattice relaxation rates. The black solid lines show the simultaneous fits of the standard model to the ^1^H data in the ordered (low-*T*) phases. The vertical bars indicate the phase transition temperatures for Na_2_B_12_H_12_ (blue) and NaCB_11_H_12_ (green).

The characteristic feature of the *closo*-dodecaborates with smaller cations (Li^+^, Na^+^) is the occurrence of high-temperature structural phase transitions [68,69], resulting in the expansion of the unit cell volume, orientational disorder for the [B_12_H_12_]^2−^ anions and crystallographic disorder for the cations. For Na_2_B_12_H_12_, the order–disorder phase transition from the low-*T* monoclinic phase to the high-*T* cubic phase is observed near 520 K [69]. NMR measurements of the ^1^H and ^23^Na spin–lattice relaxation rates in this compound have revealed that the transition from the low-*T* phase to the high-*T* phase is accompanied by the dramatic acceleration of both the reorientational motion of the anions and the translational diffusion of Na^+^ cations [17]. Figure 3 shows the behavior of the ^1^H and ^23^Na spin–lattice relaxation rates in Na_2_B_12_H_12_ in the region of the order–disorder phase transition. In the low-*T* phase, $R_{1}^{H}(T)$ exhibits the frequency-dependent maximum near 470 K that can be described in terms of the standard model at two resonance frequencies simultaneously. However, the phase transition leads to the abrupt two-orders-of-magnitude drop of the ^1^H spin–lattice relaxation rate (see Figure 3). Since, at the high-temperature slope of the relaxation rate peak, $R_{1}^{H}$ is proportional to *τ*, the observed drop of $R_{1}^{H}$ corresponds to the two-orders-of-magnitude increase in the anion reorientational jump rate *τ*^−1^. The behavior of the ^23^Na spin–lattice relaxation rate, $R_{1}^{Na}$, in the region of the phase transition is also quite unusual. As can be seen from Figure 3, the transition leads to the jump in $R_{1}^{Na}$ and to the change of the sign of its temperature dependence. Such a behavior can be described as “folding” of the relaxation rate peak [70]: because of the abrupt increase in the cation jump rate at the phase transition, $R_{1}^{Na}$ jumps directly from the low-temperature slope of the peak to its high-temperature slope. Furthermore, the ^23^Na NMR spectrum in the high-*T* phase becomes very narrow (0.31 kHz full width at half-maximum [17]); this indicates that the ^23^Na NMR parameters are governed by translational diffusion of Na^+^ ions. The slopes of the $R_{1}^{Na}(T)$ data in Figure 3 below and above the transition point yield the corresponding activation energies for diffusive Na^+^ jumps; these activation energies are included in Table 2. Since the ^23^Na spin–lattice relaxation peak is “folded”, the absolute values of the diffusive jump rates, $\tau_{d}^{-1}$, cannot be determined from the $R_{1}^{Na}(T)$ data; however, it is evident that, just above the transition point in Na_2_B_12_H_12_, the Na^+^ jump rate exceeds *ω* ≈ 2 × 10^8^ s^−1^. As mentioned in the Introduction, this result has initiated the ionic conductivity measurements leading to the discovery of the exceptional superionic conductivity in the high-*T* disordered phase of Na_2_B_12_H_12_ (~0.1 S/cm [4]).

The abrupt acceleration of anion reorientations in the disordered phase of Na_2_B_12_H_12_ has been confirmed by QENS experiments [72]. The *Q* dependence of the QENS spectra for this phase is consistent with the reorientational mechanism dominated by small-angle jumps around a single symmetry axis [72]. For Li_2_B_12_H_12_, the order–disorder phase transition is observed near 600 K [68,69]. As in the case of Na_2_B_12_H_12_, the transition from the low-*T* to the high-*T* phase of Li_2_B_12_H_12_ leads to the nearly two-orders-of-magnitude drop of the ^1^H spin–lattice relaxation rate [69], indicating the dramatic acceleration of the anion reorientations. However, the onset of the slow decomposition of Li_2_B_12_H_12_ just above the phase transition point [69] prevents any detailed studies of the dynamical properties of the disordered phase of this compound.

For practical applications, it is crucial to retain high ionic conductivity at room temperature or slightly above it. While Na_2_B_12_H_12_ exhibits the extremely high ionic conductivity above the order–disorder transition point (~520 K), at room temperature it is in the ordered phase state with much lower conductivity (~10^−7^ S/cm [4]). Therefore, it is important to reduce the temperature of the order–disorder transition, stabilizing the disordered phase state at lower temperatures. In recent years, several approaches have been used to reach this goal. One such approach is based on chemical modification of the anions. When the nearly spherical (icosahedral) [B_12_H_12_]^2−^ anion is replaced by the ellipsoidal-shaped (bicapped-square-antiprismatic) [B_10_H_10_]^2−^ anion, the transition temperature is found to be strongly reduced: Na_2_B_10_H_10_ undergoes the transition from the ordered monoclinic phase to the disordered cubic phase near 370 K [5,73]. This transition is accompanied by the characteristic “folding” of the ^23^Na relaxation rate peak [5], and the activation energies for Na^+^ diffusion resulting from the ^23^Na relaxation data in the ordered and disordered phases are included in Table 2.

**Figure 4:**
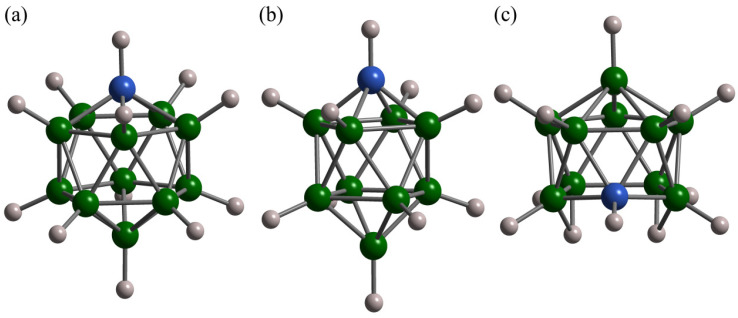
Schematic view of carbon-substituted hydroborate anions: (**a**) icosahedral *closo*-[CB_11_H_12_]^−^, (**b**) bicapped-square-antiprismatic *closo*-[1-CB_9_H_10_], and (**c**) nest-like *nido*-[7-CB_10_H_13_]^−^. Green spheres: B atoms, blue spheres: C atoms, and gray spheres: H atoms.

The next step is related to a partial carbon substitution in *closo*-hydroborate anions, when one {B–H} vertex is replaced by {C–H} [6,7]. Examples of carbon-substituted *closo*-hydroborate anions are schematically shown in Figure 4a,b. This carbon substitution leads to the anion valence change from [B_12_H_12_]^2−^ or [B_10_H_10_]^2−^ to [CB_11_H_12_]^−^ or [CB_9_H_10_]^−^. Apart from stabilizing the disordered phase state at lower temperatures, this carbon substitution also results in higher ionic conductivities in the disordered phase [6,7], presumably due to weaker anion–cation Coulombic interactions. The behavior of the ^1^H and ^23^Na spin–lattice relaxation rates in the region of the order–disorder phase transition for NaCB_11_H_12_ is shown in Figure 3, where it is compared with the corresponding behavior for NaB_12_H_12_. As can be seen from this figure, the main effects of the phase transition are similar for both compounds: the abrupt drop of the ^1^H spin–lattice relaxation rate (indicating the acceleration of anion reorientations) and the “folding” of the ^23^Na relaxation rate peak (indicating the acceleration of cation diffusion). The most impressive reduction of the phase transition temperature has been found for NaCB_9_H_10_, where the disordered phase state with the ionic conductivity of 0.03 S/cm can be retained at cooling down to room temperature [7]. The activation energies for anion reorientations and cation diffusion derived from the experimental studies of compounds with the [CB_11_H_12_]^−^ and [CB_9_H_10_]^−^ anions are included in Table 1 and Table 2. It is interesting to note that, in addition to lithium and sodium carba-*closo*-hydroborates, the order–disorder phase transition accompanied by the abrupt acceleration of anion reorientations and the increase in ionic conductivity has been found for potassium-based KCB_11_H_12_ near 340 K [76].

Before considering other approaches to stabilizing the disordered phase state, it should be mentioned that investigations of the basic properties of hydroborate salts have been recently extended to include *nido*-type hydroborates [80]. For *nido*-type anions, one of the regular 12 vertices forming the icosahedral cage of *closo*-type anions is removed, so that the resulting nest-like form of *nido* anions contains 11 vertices. As an example, Figure 4c shows the carbon-substituted *nido*-hydroborate anion [7-CB_10_H_13_]^−^. It has been found that the main properties of sodium *nido*-hydroborates resemble those of the *closo*-type counterparts. In particular, sodium *nido*-hydroborates and carbon-substituted *nido*-hydroborates also exhibit order–disorder phase transitions accompanied by the abrupt increase in ionic conductivity [80]. Furthermore, recent NMR and QENS experiments [77,80] have revealed fast reorientations of the *nido*-type anions that are strongly accelerated above the order–disorder phase transition. Note that a priori it was not evident whether the *nido*-type anions can participate in the fast reorientational motion, since they are less symmetric than the *closo*-type counterparts. The activation energies for anion reorientations derived from the ^1^H spin–lattice relaxation measurements in the ordered and disordered phases of *nido*-Na-7-CB_10_H_13_ [77] are included in Table 1. The activation energies for translational diffusion of Na^+^ ions in this compound have been determined from the $R_{1}^{Na}(T)$ measurements (for both the ordered and disordered phases) and from the PFG-NMR measurements of Na^+^ diffusivity (for the disordered phase) [77]; the resulting values are included in Table 2.

Another approach to stabilizing the disordered phase state of hydroborates is based on the crystallite size reduction caused by the mechanical milling. It has been found that ball-milling leads to the room-temperature stabilization of the high-*T*-like disordered phases for Li_2_B_12_H_12_, Li_2_B_10_H_10_, LiCB_11_H_12_, Na_2_B_12_H_12_, Na_2_B_10_H_10_, and NaCB_11_H_12_ [81]. Although these ball-milled compounds exhibit superionic conductivities at room temperature, they are usually in the two-phase state, where the disordered phases coexist with the ordered ones [81]. Furthermore, such a two-phase state is not always stable with respect to thermal cycling. 

**Figure 5:**
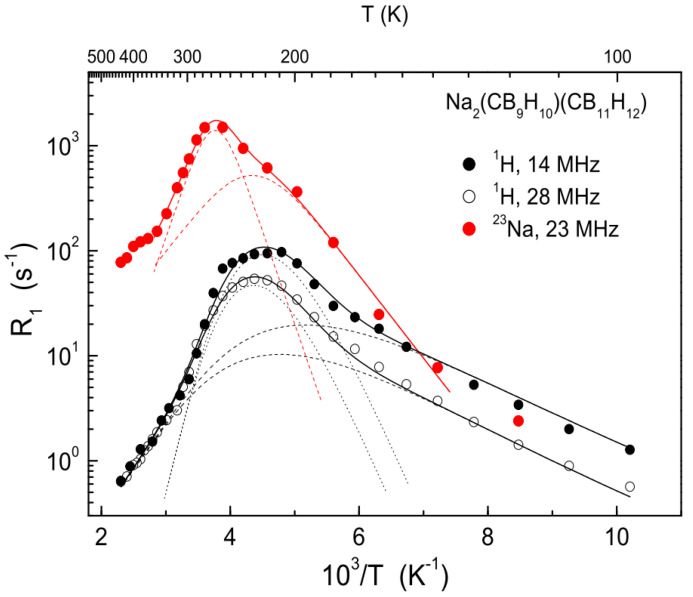
Proton spin–lattice relaxation rates measured at 14 and 28 MHz and ^23^Na spin–lattice relaxation rates measured at 23 MHz as functions of the inverse temperature for the mixed-anion solid solution Na_2_(CB_9_H_10_) (CB_11_H_12_). The black solid lines show the simultaneous fit of the two-peak model with Gaussian distributions of the activation energies to the ^1^H spin–lattice relaxation data in the range 98–435 K; the black dashed and dotted lines represent the contributions of the two components. The red solid line shows the fit of the two-peak model to the ^23^Na spin–lattice relaxation data in the range 138–349 K; the red dashed lines represent the contributions of the two components. Reprinted with permission from reference [79]. Copyright 2019 Elsevier.

The most effective approach to stabilizing the disordered phase state is based on the synthesis of mixed-anion solid solutions. It has been revealed [82,83] that the order–disorder phase transition is suppressed in mixed-anion solid solutions combining nearly spherical (icosahedral) anions, such as [B_12_H_12_]^2−^ or [CB_11_H_12_]^−^, and ellipsoidal (bicapped-square-antiprismatic) anions, such as [B_10_H_10_]^2−^ or [CB_9_H_10_]^−^. These solid solutions are found to retain the disordered state with high ionic conductivity down to low temperatures. Similar behavior has also been observed for mixed-anion solid solutions combining the nearly spherical monovalent [CB_11_H_12_]^−^ and divalent [B_12_H_12_]^2−^ anions, Na_2-*x*_(CB_11_H_12_)*_x_*(B_12_H_12_)_1-*x*_ [84], as well as those combining the *nido*- and *closo*-type anions [11]. While it is possible to prepare mixed-anion solid solutions by ball-milling the appropriate hydroborate mixtures, the cleanest and most elegant method of their synthesis is based on drying the aqueous solutions of such mixtures [82]. The mixed-anion solid solution Na_2_(CB_9_H_10_) (CB_11_H_12_) is found to exhibit the highest room-temperature ionic conductivity (~0.07 S/cm [82]) among all the studied Na-ion and Li-ion conductors. Proton spin–lattice relaxation measurements [82] have shown that in solid solutions Na_2_(CB_9_H_10_) (CB_11_H_12_) and Li_2_(CB_9_H_10_) (CB_11_H_12_) the fast reorientational motion is retained down to low temperatures. Furthermore, the suppression of the order–disorder transition in these solid solutions gives an opportunity to compare the reorientational jump rates $\tau^{-1}$ of the anions with the diffusive jump rates $\tau_{d}^{-1}$ of the cations. Figure 5 shows the behavior of both $R_{1}^{H}$ (at two resonance frequencies) and $R_{1}^{Na}$ for Na_2_(CB_9_H_10_) (CB_11_H_12_) [79].

As can be seen from this figure, due to the absence of the phase transition in the solid-solution sample, there is no “folding” of the ^23^Na relaxation rate peak; instead, a regular $R_{1}^{Na}(T)$ maximum is observed. This maximum is shifted to a somewhat higher temperature with respect to the $R_{1}^{H}(T)$ maximum, indicating that, in the region of the peaks, the diffusive motion of Na^+^ ions is slower than the reorientational motion of the anions. On the basis of the fits to the spin–lattice relaxation rate data [79], at 273 K, the diffusive jump rate $\tau_{d}^{-1}$ is about 3 × 10^8^ s^−1^, while the corresponding jump rates $\tau^{-1}$ for two reorientational processes are about 7 × 10^9^ s^−1^ and 2 × 10^9^ s^−1^. Thus, the difference between $\tau_{d}^{-1}$ and $\tau^{-1}$ is not very large, which is consistent with the conclusions [20,21] that anion dynamics can contribute to facilitating the cation diffusion. 

Table 1 summarizes the activation energies for anion reorientations, including the detailed microscopic results for [B_10_H_10_]^2−^, [CB_9_H_10_]^−^, and [CB_11_H_12_]^−^ anions [71,74,75], and the mixed-anion [B_12_H_12_]^2−^/[B_10_H_10_]^2−^ solid solution [78]. For compounds exhibiting the order–disorder phase transition, the activation energies in the disordered (high-*T*) phases are found to be considerably lower than those in the ordered (low-*T*) phases. Typical values of *E*_a_ derived from the proton NMR measurements in the disordered phases are in the range of 170–300 meV. Low energy barriers for anion reorientations in the disordered phases are consistent with very fast reorientational motion in these phases. For the most extensively studied Na salts, the activation energy in the disordered phase decreases, when [B_12_H_12_]^2−^ anions are replaced by [B_10_H_10_]^2−^ and [CB_11_H_12_]^−^ anions. For the disordered phase of Na_2_B_12_H_12_, the activation energies derived from NMR and QENS experiments are in good agreement with each other. However, for the disordered phases of other studied hydroborates, the activation energies obtained from QENS measurements appear to be lower than those resulting from the proton NMR experiments (see Table 1). As explained in [85], the most probable reason for such a systematic discrepancy is the presence of a certain distribution of H jump rates. While for proton spin–lattice relaxation measurements over wide ranges of temperature and the resonance frequency, the effects of this distribution can be easily modeled [37], it is very difficult to detect the presence of a jump rate distribution from the shape of QENS spectra. If a broad distribution is present, the standard analysis of QENS spectra tends to underestimate the changes in the quasielastic line width with temperature. Indeed, the faster part of the distribution may be outside the frequency “window” of the neutron spectrometer (contributing only to the flat background of QENS spectra), and the slower part of the distribution may be below the spectrometer resolution (contributing only to the elastic line of QENS spectra). As the temperature changes, different parts of the H jump rate distribution may appear within the frequency “window” of the neutron spectrometer; this is expected to smear the actual temperature dependence of the most probable *τ*^−1^ value. 

Table 2 summarizes the activation energies for diffusive cation (Li^+^ and Na^+^) motion in *closo*- and *nido*-hydroborate salts. For compounds showing the order–disorder phase transition, the activation energies in the disordered phases are found to be lower than those for the ordered phases; this is consistent with the acceleration of cation mobility in the disordered phases. Recently, the first direct measurements of the cation diffusion coefficients *D* using the PFG-NMR technique have been reported [9,77]. For compounds where the activation energies for both *D* and the cation jump rate have been measured (Na-7-CB_10_H_13_ and Na_2_(CB_9_H_10_)(CB_11_H_12_) [77]), these activation energies are close to each other (see Table 2). This justifies the estimates of the elementary jump length *L* based on comparison of the values of *D* and the cation jump rate. For Na_2_(CB_9_H_10_) (CB_11_H_12_), such a straightforward estimate of *L* at 330 K yields 6.9 Å [77], which is considerably larger than the distance between the nearest-neighbor tetrahedral and octahedral Na^+^ sites in the hexagonal close-packed lattice of this compound, *d*_TO_ = 4.28 Å [79]. A possible reason for the large effective *L* value may be related to correlations between jumps of different cations adjoining a large anion [21], when a rotation of the single anion can facilitate jumps of several cations.

According to the Nernst-Einstein equation, the ionic conductivity *σ* is proportional to *D*/*T*. Therefore, one may expect that the activation energies for *D* (or the cation jump rate) and for the product *σT* are the same. However, for most of the studied *closo*-hydroborates, the measured activation energies for *D* and the cation jump rate appear to be considerably lower than those for *σT* [5,7,71,77]. The higher activation energies for the measured conductivity may presumably be attributed to the effects of large-scale inhomogeneities (such as grain boundaries) [5,86].

## 5. Effects of Nanoconfinement on Dynamical Properties of Hydroborates 

Another promising approach to modifying the dynamical properties of hydroborate salts is based on their confinement in nanoporous hosts. Generally, such a confinement leads to a suppression of the long-range order in the confined material and, in many cases, to an enhancement of the mobility of anions and/or cations. Most of the studies in this direction have been performed on lithium borohydride infiltrated into nanoporous carbon or SiO_2_ hosts [24,87,88,89,90,91,92,93,94,95,96]. The characteristic feature of these nanocomposites is a coexistence of two distinct fractions of nanoconfined LiBH_4_. One of these fractions shows the dynamical behavior similar to that of bulk LiBH_4_, whereas the other fraction exhibits very high mobilities of both the anions and the cations. In NMR experiments, this is manifested as a coexistence of broad and narrow components in the ^1^H and ^7^Li NMR spectra. Since the fraction of mobile spins has been found to grow with decreasing pore size, the mobile fraction should be attributed to the regions adjoining (within a few nanometers) the pore walls, while the slow fraction is located in the core of the pores. The mobile fraction of the nanoconfined material appears to be responsible for the high ionic conductivity of nanoconfined LiBH_4_ at room temperature [93,95]. However, systematic studies of the effects of pore size on the ionic conductivity of nanoconfined hydroborate salts have not been reported so far. It is interesting to note that, apart from the enhanced reorientational jump rate of BH_4_^−^ anions in the mobile fraction [90,91,92,95], these anions also participate in translational diffusion at the frequency scale of 10^5^ s^−1^ or higher. Indeed, the narrow component of the ^1^H NMR spectra has a width of about 1 kHz [24,92,94], and the reorientational motion alone cannot lead to such a narrowing (see Section 2). Experiments at various temperatures have revealed that the fraction of mobile spins increases with temperature [24,92,94,96]. This suggests that there is no fixed interface between the two fractions, which is consistent with the presence of broad continuous distributions of the jump rates [94].

It should be noted that most of the studies of dynamical properties of nanoconfined LiBH_4_ rely only on NMR lineshape measurements; these measurements can only distinguish between slow and fast motions at the frequency scale of ~10^5^ s^−1^, without giving more specific information on the atomic jump rates. Nuclear spin relaxation experiments on nanoconfined systems are still quite rare. Recently, for LiBH_4_ confined in nanoporous carbon, the frequency dependence of the ^1^H spin–lattice relaxation rate over the very wide range from 50 kHz to 20 MHz has been studied by the field-cycling technique [94]. It has been found that at *T* = 298 K and 371 K (where the ^1^H relaxation rate is governed by Li^+^ jumps via ^1^H–^7^Li dipole–dipole interactions), $R_{1}^{H}$ is proportional to *ω*^−1^; this suggests the presence of a broad distribution of Li^+^ jump rates. Furthermore, the exchange of the anions between the two fractions of LiBH_4_ confined in nanoporous carbon has been studied by the selective inversion of the narrow component of the ^1^H NMR spectrum [94]; the exchange time constant of the order of 5 ms appears to be nearly temperature-independent over the range of 280–420 K. On the other hand, for LiBH_4_ confined in nanoporous SiO_2_ (SBA-15), the fast exchange of Li^+^ cations between the two fractions has been observed only above the order–disorder (orthorhombic-hexagonal) phase transition temperature (~370 K) of the bulk-like LiBH_4_ fraction [96]. It should be kept in mind that the detailed microscopic picture of motions in nanoconfined LiBH_4_ may also depend on the material of nanoporous scaffold (carbon or SiO_2_) and on the shape of the pores.

Recent studies of combined effects of anion substitution and nanoconfinement [97] have shown that, for mixed-anion systems Li(BH_4_)*_x_*I_1-*x*_ and Li(BH_4_)*_x_*(NH_2_)_1-*x*_ confined in nanoporous SiO_2_ or Al_2_O_3_, the Li^+^ conductivity at room temperature is higher than both the conductivities of the corresponding bulk materials and the conductivity of nanoconfined LiBH_4_. These results demonstrate that combining partial anion substitution and nanoconfinement may be a promising approach to reach high ionic conductivity at room temperature in complex hydrides.

The first synthesis of nanoconfined *closo*-hydroborate has been reported by Yan et al. [98] using nanoporous SiO_2_ (SBA-15 with cylindrical pores of an average diameter of 5.9 nm) as a scaffold. The ^7^Li NMR spectrum of the resulting Li_2_B_12_H_12_/SBA-15 nanocomposite is found to consist of a narrow (1.1 kHz) and broader (~6.5 kHz) components which do not show any significant changes over the temperature range from 298 K to 378 K. This indicates a coexistence of temperature-independent fractions of more mobile and less mobile Li^+^ ions. According to X-ray diffraction measurements, the dominant phase of the Li_2_B_12_H_12_/SBA-15 nanocomposite corresponds to the ordered (low-*T*) phase of bulk Li_2_B_12_H_12_, and the room-temperature ionic conductivity (8.3 × 10^−6^ S/cm [98]) is quite modest.

**Figure 6:**
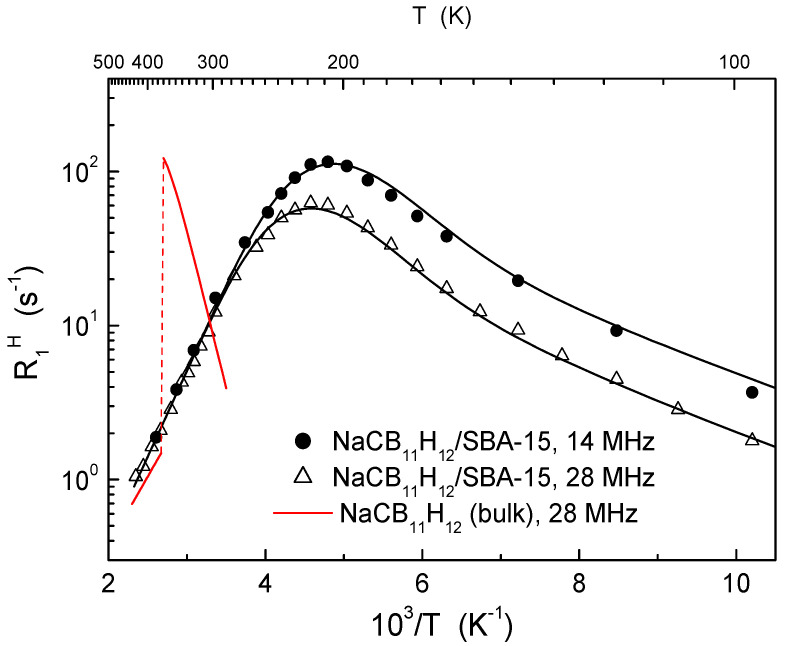
Proton spin–lattice relaxation rates measured at the resonance frequencies of 14 and 28 MHz in the nanoconfined NaCB_11_H_12_/SBA-15 system (average pore diameter of 8 nm) [99]. The black solid lines show the simultaneous fit of the two-peak model to the data. For comparison, the red line shows the behavior of the proton spin–lattice relaxation rate in bulk NaCB_11_H_12_ [71].

The remarkable enhancement of both the reorientational motion of the anions and the translational diffusion of the cations has been recently revealed in the carbon-substituted *closo*-hydroborate NaCB_11_H_12_ confined in SBA-15 with the average pore diameter of 8 nm [99]. As can be seen from Figure 6, the behavior of $R_{1}^{H}(T)$ in nanoconfined NaCB_11_H_12_/SBA-15 strongly differs from that observed for the bulk NaCB_11_H_12_ [71]. In particular, the order–disorder phase transition in the bulk material (near 376 K) is suppressed in the nanocomposite, so that the disordered phase with high reorientational mobility is retained down to low temperatures. In contrast to the case of nanoconfined LiBH_4_, the proton NMR spectra in NaCB_11_H_12_/SBA-15 do not exhibit a two-component structure with a very narrow component. Thus, there are no signs of fast translational anion mobility in NaCB_11_H_12_/SBA-15. However, Na^+^ cations in this system are found to participate in the fast translational diffusion; this is supported by the strong ^23^Na NMR line narrowing near 180 K leading to the plateau line width values of about 1 kHz and the ^23^Na spin–lattice relaxation rate maximum near 270 K [99]. These results suggest that nanoconfinement of *closo*-hydroborates may be an effective way to reach high ionic conductivity at room temperature.

## 6. Conclusions and Outlook

This brief review emphasizes the potential of NMR for the investigation of a rich picture of anion and cation dynamics in polyhydroborate salts. The discoveries of high Li^+^ conductivity in LiBH_4_ [100] and high Li^+^ and Na^+^ conductivities in *closo*-hydroborates (initiated by NMR results for Na_2_B_12_H_12_ [17]) have shifted the focus of interest to this class of materials from the hydrogen storage properties to the properties useful for solid electrolytes in rechargeable batteries. Studies of the key factors governing the performance of polyhydroborate salts as solid electrolytes are of great importance for future developments.

One of such factors is the relation between anion reorientations and cation translational mobility. For most of the studied compounds, fast cation diffusion is observed in the phases exhibiting extremely fast anion reorientations. Although this relation in *closo*-hydroborates has been addressed in a number of ab initio calculations [18,19,20,21], detailed experimental studies of the mechanisms of cation diffusion are still lacking. The unusual mechanism of ammonia-assisted Li^+^ diffusion in the new borohydride-ammine LiBH_4_·1/2NH_3_ has been suggested recently on the basis of structural studies and ab initio calculations [101]: an interstitial Li^+^ ion “borrows” an NH_3_ molecule from the framework Li^+^ to perform a jump, and then delivers this molecule back to the framework Li^+^. In order to verify this mechanism, it would be interesting to probe the corresponding displacements of NH_3_ molecules by NMR measurements.

Another important factor is the interaction of hydroborate salts with the wall surfaces in nanoporous materials. Understanding the nature of the mobility enhancement near the surfaces may contribute to the search for new materials with high ionic conductivities.

## Figures and Tables

**Table 1:** Activation energies for anion reorientations in *closo*- and *nido*-hydroborate salts, as derived from NMR and QENS experiments.

Compound	Activation Energy (meV)	*T* Range (K)	Method	Reference
Li_2_B_12_H_12_ (LT phase)	1400 (50)	490–590	NMR	[69]
Na_2_B_12_H_12_ (LT phase)	770 (20)	400–520	NMR	[17]
Na_2_B_12_H_12_ (HT phase)	270 (40)	523–570	NMR	[17]
	259 (22)	480–620	QENS	[72]
K_2_B_12_H_12_	1070 (54)	270–370	NMR ^a^	[65]
	800 (8)	366–564	NMR	[17]
Rb_2_B_12_H_12_	910 (46)	220–340	NMR ^a^	[65]
	549 (5)	315–560	NMR	[17]
(NH_4_)_2_B_12_H_12_	930 (40)	250–350	NMR ^a^	[65]
	486 (8)	297–474	NMR	[67]
Cs_2_B_12_H_12_	600 (30)	180–300	NMR ^a^	[65]
	333 (15)	430–530	QENS	[66]
	427 (4)	260–570	NMR	[17]
Na_2_B_10_H_10_ (HT phase)	180 (30)	375–435	NMR	[74]
	124 (2)	365–500	QENS	[74]
Rb_2_B_10_H_10_	522 (7) ^b^	219–573	NMR	[75]
	288 (3) and 197 (2) ^c^	400–680	QENS	[75]
LiCB_11_H_12_ (LT phase)	409 (11)	278–384	NMR	[71]
LiCB_11_H_12_ (HT phase)	177 (7)	390–435	NMR	[71]
NaCB_11_H_12_ (LT phase)	409 (7)	278–376	NMR	[71]
NaCB_11_H_12_ (HT phase)	177 (8)	380–435	NMR	[71]
KCB_11_H_12_ (LT phase)	330 (20)	258–332	NMR	[76]
KCB_11_H_12_ (HT phase)	191 (4)	341–435	NMR	[76]
	149 (2)	345–470	QENS	[76]
LiCB_9_H_10_ (LT phase)	302 (7) ^b^	258–332	NMR	[74]
LiCB_9_H_10_ (HT phase)	299 (5)	359–418	NMR	[74]
	170 (2)	330–410	QENS	[74]
NaCB_9_H_10_ (LT phase)	234 (6) ^b^	179–278	NMR	[74]
NaCB_9_H_10_ (HT phase)	205 (5)	287–376	NMR	[74]
	130 (2)	290–445	QENS	[74]
Na-7-CB_10_H_13_ (LT phase)	330 (20)	209–315	NMR	[77]
Na-7-CB_10_H_13_ (HT phase)	219 (5)	332–384	NMR	[77]
Na_2_(B_12_H_12_)_0.5_(B_10_H_10_)_0.5_	240 (10)	300–390	QENS	[78]
Na_2_(CB_9_H_10_)(CB_11_H_12_)	430 (40) and 147 (9) ^c^	278–376	NMR	[79]

^a^ From ^11^B line width measurements; ^b^ average values for activation energy distributions; ^c^ for the slow and fast jump processes, respectively.

**Table 2:** Activation energies for translational diffusion of Li^+^ and Na^+^ ions in *closo*- and *nido*-hydroborate salts, as derived from NMR and PFG-NMR experiments.

Compound	Activation Energy (meV)	*T* Range (K)	Method	Reference
Na_2_B_12_H_12_ (LT phase)	450 (10)	458–517	NMR	[17]
Na_2_B_12_H_12_ (HT phase)	410 (25)	523–580	NMR	[17]
Na_2_B_10_H_10_ (LT phase)	750 (20)	298–367	NMR	[5]
Na_2_B_10_H_10_ (HT phase)	190 (10)	375–435	NMR	[5]
LiCB_11_H_12_ (LT phase)	422 (6)	332–376	NMR	[71]
LiCB_11_H_12_ (HT phase)	92 (7)	392–426	NMR	[71]
NaCB_11_H_12_ (LT phase)	327 (11)	340–367	NMR	[71]
NaCB_11_H_12_ (HT phase)	152 (8)	376–418	NMR	[43]
LiCB_9_H_10_ (HT phase)	55 (9)	358–418	NMR	[7]
NaCB_9_H_10_ (HT phase)	153 (7)	293–401	NMR	[7]
Na-7-CB_10_H_13_ (LT phase)	320 (9)	198–315	NMR	[77]
Na-7-CB_10_H_13_ (HT phase)	116 (7)	333–385	NMR	[77]
	134 (3)	320–403	PFG-NMR	[77]
Li(CB_9_H_10_)_0.7_(CB_11_H_12_)_0.3_	~295	298–333	PFG-NMR	[9]
Na_2_(B_12_H_12_)_0.5_(B_10_H_10_)_0.5_	240 (10)	180–320	NMR	[78]
Na_2_(CB_9_H_10_)(CB_11_H_12_)	353 (11)	138–349	NMR	[79]
	135 (8)	350–435	NMR	[79]
	118 (1)	298–403	PFG-NMR	[77]
